# Preliminary Study on the Pathogenic Mechanism of Jujube Flower Disease in Honeybees (*Apis mellifera ligustica*) Based on Midgut Transcriptomics

**DOI:** 10.3390/genes15050533

**Published:** 2024-04-24

**Authors:** Yali Du, Kai Xu, Huiting Zhao, Ying Wu, Haibin Jiang, Jinming He, Yusuo Jiang

**Affiliations:** 1 College of Animal Science, Shanxi Agricultural University, Jinzhong 030801, China; duyali2000@yeah.net; 2 Apiculture Science Institute of Jilin Province, Jilin 132108, China; xukaiyuzhong@126.com (K.X.); wu569703@163.com (Y.W.); jhb18047513706@163.com (H.J.); hejinmingmifeng@163.com (J.H.); 3 College of Life Science, Shanxi Agricultural University, Jinzhong 030801, China; zhaohting@126.com

**Keywords:** honeybees, jujube flower disease, histomorphology, enzyme activity, transcriptomics

## Abstract

Honeybees are prone to poisoning, also known as jujube flower disease, after collecting nectar from jujube flowers, resulting in the tumultuous demise of foragers. The prevalence of jujube flower disease has become one of the main factors affecting the development of the jujube and beekeeping industries in Northern China. However, the pathogenic mechanisms underlying jujube flower disease in honeybees are poorly understood. Herein, we first conducted morphological observations of the midgut using HE-staining and found that jujube flower disease-affected honeybees displayed midgut damage with peritrophic membrane detachment. Jujube flower disease was found to increase the activity of chitinase and carboxylesterase (CarE) and decrease the activity of superoxide dismutase (SOD), catalase (CAT), glutathione S-transferase (GST), and the content of CYP450 in the honeybee midgut. Transcriptomic data identified 119 differentially expressed genes in the midgut of diseased and healthy honeybees, including *CYP6a13*, *CYP6a17*, *CYP304a1*, *CYP6a14*, *AADC*, and *AGXT2*, which are associated with oxidoreductase activity and vitamin binding. In summary, collecting jujube flower nectar could reduce antioxidant and detoxification capacities of the honeybee midgut and, in more severe cases, damage the intestinal structure, suggesting that intestinal damage might be the main cause of honeybee death due to jujube nectar. This study provides new insights into the pathogenesis of jujube flower disease in honeybees.

## 1. Introduction

Jujube (*Ziziphus jujuba* Mill.), or Chinese date, is the most economically and ecologically important Rhamnaceae species. It is widespread in China, with a cultivation area of approximately two million hectare and an annual production of over eight million tons [1]. Jujube flowers are insect-pollinated and are the main nectar source in North and Northwest China because of their prolonged flowering period and adequate nectar production [2,3]. However, the phenomena of toxicity and even death commonly occur among honeybees, especially *Apis mellifera* L., during the blooming period of jujube trees. Hence, this disease, which has been termed the “jujube flower disease”, severely restricts bee breeding and colony development [2,4,5]. However, due to this disease, the enthusiasm of beekeepers to use jujube nectar has greatly decreased. In the north of China, some beekeepers prefer to feed a colony rather than allow the honeybees to collect jujube nectar to prevent the development of this disease, which may prove fatal to the honeybees. The prevalence of jujube flower disease is one of the main factors affecting the development of the Chinese jujube and beekeeping industries.

Since the 1950s and 1960s, beekeepers have mostly deduced the causes of jujube flower disease based on their experience of raising and managing honeybees. Zhang [6] surveyed bee colonies in the Luliang area of Shanxi Province during the jujube flowering period and found that in dry climatic conditions with a strong southwest wind and a severe shortage of water, the incidence of jujube flower disease was high, and the bee population decreased by 30–50%; therefore, they postulated that jujube flower disease may be caused by severe drought and heat. Subsequently, based on investigations into the incidence of jujube flower disease in the Zhongmou area of Henan Province, Xi [7] concluded that it is neither an infectious disease nor pollen poisoning. Rather, they attributed the disease to the combination of a dry climate, thick nectar, and overworking of the foragers. In the 1990s, researchers explained the pathogenesis of jujube flower disease based on the chemical composition of jujube honey. Li and Fan [8] compared the compositional differences in sugar, alkaloid, mineral, and toxic contents in jujube and robinia honey, and found that the potassium content of jujube honey was 0.141%, which was more than 16 times that of robinia honey, suggesting that excessive amounts of potassium in jujube honey may be the main cause of bee poisoning. In addition, the high alkaloid content has been cited as the main cause of jujube flower disease [9]. With the rapid development of high-throughput sequencing technology, in-depth studies on jujube flower disease have been conducted. Using amplicon sequencing of 16 S rRNA, the intestinal microbial diversity of healthy honeybees was compared with that of honeybees affected by jujube flower disease, and the abundance of Firmicutes and Actinobacteria in the midgut and hindgut of diseased honeybees was found to be significantly lower than that in healthy honeybees [10]. According to metabolomic detection using liquid chromatography-mass spectrometry (LC-MS) and gas chromatography-mass spectrometry (GC-MS) non-targeted metabolomics, 73 differential metabolites were enriched in carbohydrate metabolism, and further KEGG enrichment analysis showed that carbohydrate metabolism in the honeybee midgut was disturbed after the collection of jujube flower nectar [11]. However, currently, there are no reports on the pathogenesis of jujube flower disease at the transcriptomic level.

As the main tissue involved in insect digestion and detoxification, the midgut plays an important role in insect feeding, digestion, growth, development, and defense against exogenous toxic substances. Here, we first observed the morphological structure and measured the enzyme activity of the honeybee midgut, and then compared the gene expression between healthy honeybees and those affected by jujube flower disease based on midgut transcriptomics. We believe that these results will provide a theoretical basis for exploring the pathogenesis and prevention of jujube flower disease.

## 2. Materials and Methods

### 2.1. Tested Honeybee

The experimental bee species used was the Italian bee, *Apis mellifera ligustica*. Before the jujube flowering season (approximately 15 May), 12 healthy and disease-free bee colonies were placed in a jujube forest with very little honey and powder source around a 5 km radius in Nanguang village (37°23′2.00″ N, 112°31′12.04″ E), Northern China. During the jujube blooming period (8–15 June), when the occurrence of jujube flower disease was severe, sick bees with the following symptoms were randomly collected from the hive entrances: abdominal contraction, slow response, loss of flying ability, jumping, and crawling. These bees were assigned to the diseased group (DG). Simultaneously, healthy foragers collecting jujube nectar from flowers were randomly selected, and if they were agile and their abdomens were healthy and shiny, they were assigned to the healthy group (HG). The bee samples were quickly returned to the laboratory in a wooden box (20 × 8 cm^2^), and the body surface of the bee was sequentially rinsed with distilled water and 75% alcohol after application of freezing anesthesia. Using tweezers sterilized with 75% alcohol, the gut of the honeybee was extracted by holding the ventral stalk and stinger or the seventh ventral segment. The midgut was cut off, placed in phosphate buffer solution (PBS) at 4 °C, and then dried with an absorbent paper. One part of the dried midgut was individually placed in 4% paraformaldehyde fixing solution for morphology and tissue observation, while the other part of the midgut was quickly placed in liquid nitrogen, and then stored at −80 °C for enzyme activity assays, RNA sequencing (RNA-Seq), and quantitative real-time polymerase chain reaction (qRT-PCR). A mixed sample of the midgut from 30 worker bees was used as a biological replicate, and 3 biological replicates were conducted on each sample.

### 2.2. Morphological Observation

Midguts fixed with 4% paraformaldehyde that were in good condition were pruned, dehydrated, embedded, sliced, stained with hematoxylin and eosin, and sealed using the methods published by Sun et al. [12]. The midgut tissue morphology was observed using a panoramic scanner (3D HISTECH, Budapest, Hungary).

### 2.3. Enzyme Activity Assay

The frozen samples from HG and DG were placed in a 1.5 mL centrifuge tube and maintained at 4 °C until melting. After weighing, saline was added to the tubes in the proportion of 1 g:9 mL. Then, the midgut samples were homogenized under ice bath conditions and were centrifuged at 3000 r/min for 10 min. The obtained supernatant was used for enzyme activity assays. The CYP450 content was determined using the Insect CYP450 ELISA Kit (MLBIO Biotechnology Co., Ltd., Shanghai, China). The activities of five enzymes: chitinase, superoxide dismutase (SOD), catalase (CAT), glutathione S-transferase (GST), and carboxylesterase (CarE), were determined using their respective assay kits, according to the manufacturer’s instructions (Nanjing Jiancheng Bioengineering Institute, Nanjing, China). The absorbance at corresponding wavelengths was assessed using the Synergy H1^TM^ microplate reader (BioTek, Winooski, VT, USA). Each experiment was repeated with three independent replicates.

### 2.4. cDNA Library Construction and Illumina Sequencing

Total RNA from the midgut of HG and DG bees was extracted using TRIzol^TM^ reagent (Invitrogen, Carlsbad, CA, USA) and then assessed for RNA purity and concentration. Six cDNA libraries (HG1, HG2, and HG3; DG1, DG2, and DG3) were constructed using NEBNext^®^ Ultra^TM^ RNA Library Prep Kit for Illumina^®^ (NEB, Ipswich, MA, USA) following the manufacturer’s recommendations, and sequenced by Beijing Novogene Co., Ltd. (Beijing, China) on an Illumina Novaseq platform. Raw Illumina sequence reads were filtered to remove low-quality reads and reads containing adapters or poly-N. Simultaneously, Q20, Q30, and GC contents of the clean data were calculated. The clean reads were then aligned to the *A. mellifera* reference genome (Amel_HAv3.1) using Hisat2 v2.0.5. Mapped reads of each sample were assembled using StringTie (v1.3.3b) and quantified using featureCounts v1.5.0-p3. The fragments per kilobase of transcript sequence per million base pairs (FPKM) values for each gene were calculated, and genes were considered expressed if FPKM was >1. Differentially expressed genes (DEGs) between HG and DG were screened by adjusted *p*-value (padj) < 0.05 and |log_2_(FoldChange)| > 0 using the DESeq2 package in R software (v4.2.0). Gene Ontology (GO) and Kyoto Encyclopedia of Genes and Genomes (KEGG) pathway enrichment analyses of DEGs were performed using the clusterProfiler package in R software.

### 2.5. Validation of DEGs Using qRT-PCR

First-strand cDNA was synthesized from 1 μg of total RNA using the PrimeScript^TM^ RT Reagent Kit with gDNA Eraser (Perfect Real Time, TaKaRa, Dalian, China) and then diluted five times with nuclease-free water. Then, qRT-PCR analysis was performed on a 7500 Real-time PCR system (ABI, FosterCity, CA, USA) using TB Green^®^ Premix Ex Taq^TM^ (Tli RNaseH Plus, TaKaRa) in a 15 μL reaction containing 1.5 µL of cDNA, 0.6 µL of each primer (10 μM), 7.5 µL of SYBR Premix Ex Taq II (2×), 0.3 µL of ROX Reference Dye II (2×), and 4.5 μL of ddH_2_O. The procedure included a reaction at 95 °C for 30 s, followed by 45 cycles at 95 °C for 5 s, and another reaction at 62 °C for 34 s. At the end of the analysis, melt curves were generated using the following conditions: 95 °C for 15 s, 62 °C for 1 min, and 95 °C for 15 s. Three technical replicates were performed for each pooled sample. Specific primers for qRT-PCR were designed using the Primer 3.0 plus server (https://www.primer3plus.com/index.html (accessed on 6 May 2022)), and the primers used are listed in Table S1. All data obtained by qRT-PCR were analyzed using 7500 Software(v2.0), and the relative expression was normalized to *AmelArp1* (LOC406122) mRNA using the comparative 2^−ΔΔCt^ method.

### 2.6. Statistical Analyses

Significant differences in enzyme activity and mRNA expression levels were determined using an independent sample *t*-test (SPSS 25.0, IBM, New York, NY, USA). The results are reported as means ± standard error and plotted using GraphPad Prism 9.0 (GraphPad Software Inc., San Diego, CA, USA).

## 3. Results

### 3.1. Effect of Jujube Flower Disease on Honeybee Midgut Tissue

**Figure 1:**
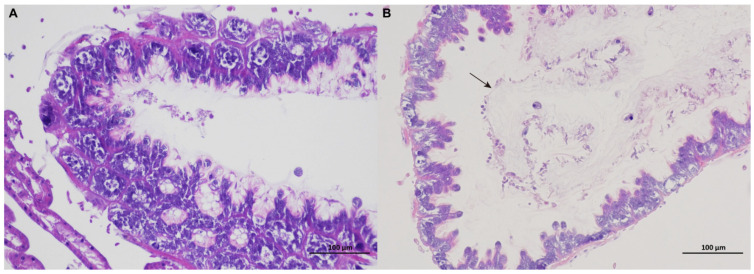
Histological image of honeybee midgut: (**A**) healthy group and (**B**) diseased group. The black arrow indicates the detached peritrophic membrane.

**Figure 2:**
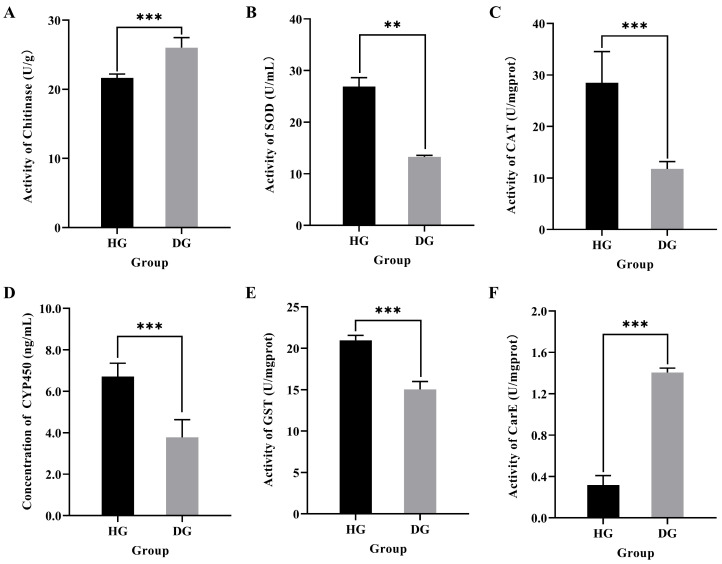
The activity of (**A**) chitinase, antioxidant enzymes (**B**) SOD and (**C**) CAT, and detoxification enzymes (**D**) CYP450, (**E**) GST, and (**F**) CarE in the honeybee midgut. Data in the graph are shown as mean ± SE. The symbol above the bar indicates a significant difference between the two groups (** *p* < 0.01; *** *p* < 0.001; *t*-test). HG and DG represent healthy groups and diseased groups, respectively.

Histopathological observations of the midgut tissue are shown in Figure 1. Hematoxylin and eosin staining showed that the intestinal peritrophic membrane in healthy honeybees was relatively complete, but in diseased honeybees, the peritrophic membrane was completely detached (black arrow in Figure 1) and the cell layer of the intestinal wall was significantly thinner. This suggests that jujube flower disease destroys the structural integrity of the honeybee midgut. In addition, chitinase activity was significantly higher in the midgut of honeybees with jujube flower disease than in that of healthy bees (*t* = −8.049, *p* < 0.001; Figure 2A).

### 3.2. Effect of Jujube Flower Disease on Antioxidant and Detoxification Capacity in Honeybee Midgut

The activities of antioxidant and detoxification enzymes in the midgut were significantly altered in honeybees affected by jujube flower disease. As shown in Figure 2, the activities of antioxidant enzymes SOD (*t* = 13.479, *p* = 0.004) and CAT (*t* = 6.578, *p* < 0.001) and detoxification enzymes CYP450 (*t* = 6.008, *p* < 0.001) and GST (*t* = 9.130, *p* < 0.001) were significantly lower in the midgut of DG bees than in those of the HG bees (Figure 2B–E), while the activity of CarE (*t* = −32.435, *p* < 0.001) was significantly higher in the midgut of DG bees compared to that in the HG bees (Figure 2F).

### 3.3. Transcriptome Analysis

Three cDNA libraries representing the midgut of healthy groups (HG1, HG2, and HG3), and three representing the diseased groups (DG1, DG2, and DG3), were constructed using the Illumina sequencing platform, and each of these libraries produced 45.78–48.17 million raw reads (Table S2). After quality filtering, HG and DG averaged 44.22 and 45.47 million clean reads, respectively. Q20 was higher than 97% and Q30 was higher than 92%. In each library, the number of tags that could be uniquely mapped to the reference genome ranged from 78.91 to 93.85%. Approximately 92.58% of the reads were observed in known exons and 4.13% were in the predicted intergenic or intron regions. Pearson’s correlation analysis revealed that the correlations among samples in the same experimental group were greater than 0.93, indicating the reliability of the RNA-Seq and sample selection. The Illumina sequencing data obtained in this study have been submitted to the Sequence Read Archive of the National Center for Biotechnology Information (accession number: PRJNA1089829).

**Figure 3:**
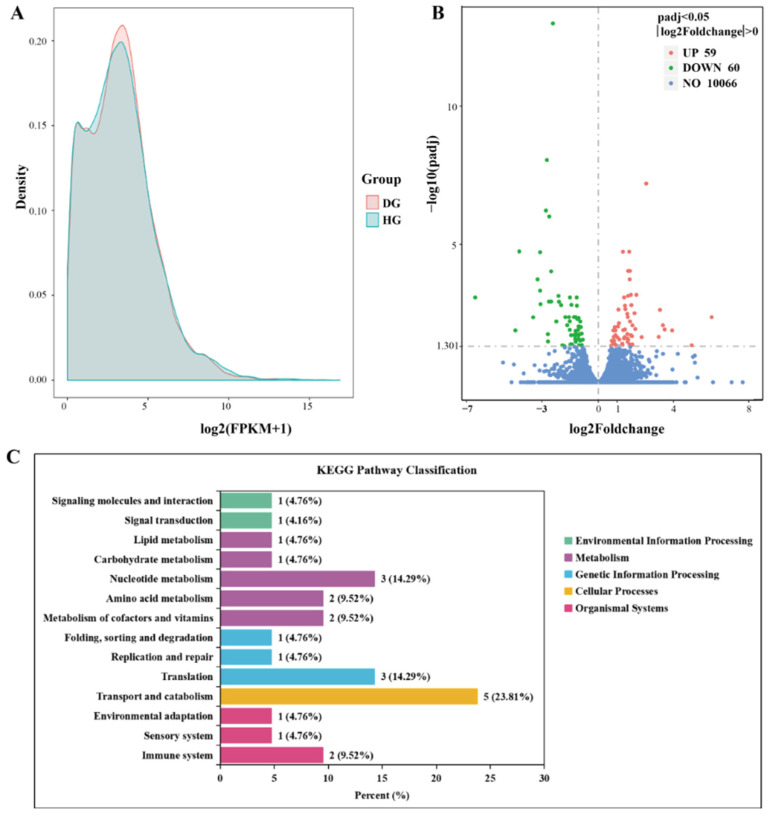
Bioinformatic analyses of RNA-Seq data. (**A**) Density distribution of FPKM for HG and DG. (**B**) Volcano plots of the differential gene expression between HG and DG. The differentially expressed genes were screened by |log2 FoldChange| > 0 and adjusted *p*-value (padj) < 0.05. (**C**) Statistical graph of KEGG functional categories of DEGs between HG and DG. The horizontal coordinate indicates the percent of genes enriched on the KEGG pathway to total DEGs, and the vertical coordinate is the name of KEGG pathway class II. The diagram distinguishes the KEGG pathway class I with different colors.

Prior to DEG analysis, FPKM distribution was used to examine sample changes between two groups. FPKM and density distribution analyses showed that most of the genes belonged to the same group (Figure 3A). There were 119 DEGs between the midguts of the two groups (Figure 3B and Table S3). Compared with healthy honeybees, the midgut of diseased honeybees contained 59 significantly upregulated and 60 significantly downregulated DEGs. Gene annotation was used to identify the DEGs between the two groups, including genes encoding the cytochrome P450 (CYP450) gene family (CYP6a13, CYP6a14, CYP6a17, and CYP304a1), and cytochrome P450 304a1 (CYP304a1), Apidaecin 1, Cuticular protein 14, Chitinase 10, and the G-protein coupled receptor Mth.

### 3.4. GO and KEGG Enrichment of DEGs

GO enrichment analysis showed that the DEGs in the midgut of healthy honeybees and those affected by jujube flower disease were distributed over 200 GO terms, involving items related to molecular function, such as oxidoreductase activity, vitamin B6 binding, ATPase activity, and cysteine peptidase activity, items related to cell composition, such as membrane composition, and those related to biological processes, such as macromolecular biosynthesis, proteolysis, stress response, and chitin metabolism (Table S4). Among these items, 10 GO terms were significantly enriched, and the DEGs involved in these functions included 4 genes related to oxidoreductase activity, *CYP6a17*, *CYP6a14*, *CYP6a13*, and *CYP304a1*, 2 genes related to vitamin B6 binding, aromatic-L-amino-acid decarboxylase (*AADC*) and alanine-glyoxylate aminotransferase 2 (*AGXT2*), and 2 genes related to cell adhesion, Vinculin and Nidogen-2 (Table 1).

Analysis of the enriched KEGG pathways revealed that a total of 21 DEGs were related to transport and catabolism, translation, nucleotide metabolism, amino acid metabolism, metabolism of cofactors and vitamins, carbohydrate metabolism, lipid metabolism, signal transduction, environmental adaptation, and immune system. Other KEGG pathways (Figure 3C) included tyrosine metabolism, nicotinic acid and nicotinamide metabolism, tryptophan metabolism, pyrimidine metabolism, cysteine and methionine metabolism, glycolysis/gluconeogenesis, and phosphoinositide metabolism, and other pathways, including the MAPK signaling pathway, Toll and Imd signaling pathway, and other signaling pathways (Table S5). Compared with the results of the GO terms, we found that two genes were the same as the DEGs annotated in the KEGG pathway: AADC enriched in tyrosine and tryptophan metabolism, and AGXT2 enriched in glycerophospholipid metabolism (Table 2).

### 3.5. Transcriptome Verification

**Figure 4:**
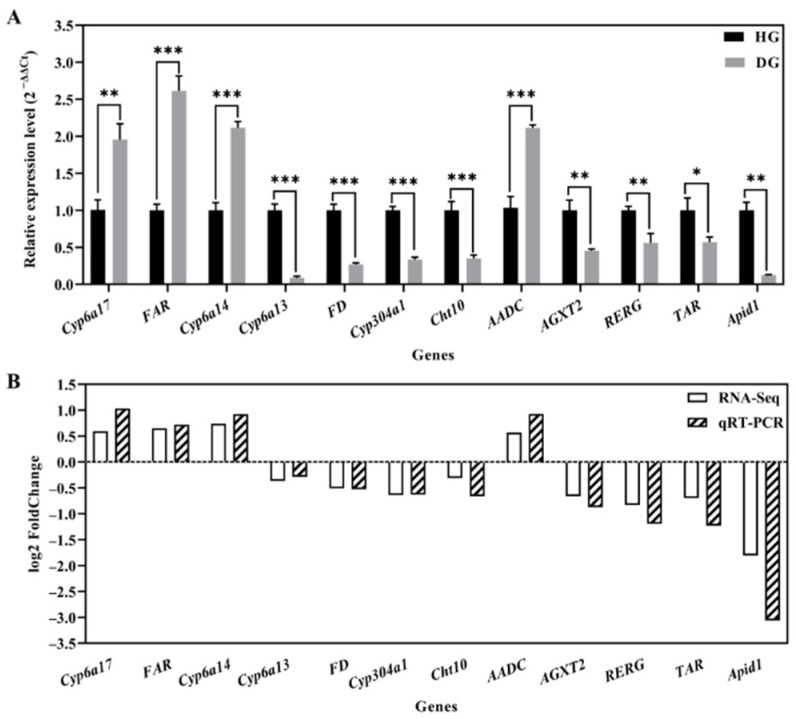
Validation of the qRT-RCR results. (**A**) Expression analysis of 12 DEGs between HG and DG evaluated by qRT-PCR. Data in the graph are shown as mean ± SE. The symbol above the bar indicates a significant difference between the two groups (* *p* < 0.05, ** *p* < 0.01; *** *p* < 0.001; *t*-test). (**B**) The expression trend of 12 DEGs between HG and DG analyzed by RNA-Seq and qRT-PCR, respectively. Expression trends are presented based on the log_2_Fold change DG/HG condition.

To verify the accuracy and credibility of the RNA-Seq results, we used real-time fluorescence quantitative PCR to compare and analyze the relative expression levels of 12 DEGs related to oxidoreductase activity, response to environmental stress, chitin metabolism, and vitamin metabolism (Figure 4). The results showed that the expressions of *CYP6a17* (*t* = −6.528, *p* = 0.003), *CYP6a14* (*t* = −14.618, *p* < 0.001), *AADC* (*t* = −11.876, *p* < 0.001), and *fatty acyl-CoA reductase* (*FAR*; *t* = −12.868, *p* < 0.001) were upregulated in the midgut of diseased bees compared to those of healthy bees, whereas the levels of *Cyp6a13* (*t* = 18.025, *p* < 0.001), *Cyp304a1* (*t* = 18.839, *p* < 0.001), *AGXT2* (*t* = 6.783, *p* = 0.002), *farnesol dehydrogenase* (*FD*; *t* = 14.882, *p* < 0.001), *Chitinase 10* (*Cht10*; *t* = 8.870, *p* < 0.001), *ras-related and estrogen-regulated growth inhibitor* (*RERG*; *t* = 5.523, *p* = 0.005), *tyramine receptor* (*TAR*; *t* = 4.149, *p* = 0.014), and *apidaecin 1* (*Apid1*; *t* = 14.161, *p* = 0.005) were downregulated in the midgut of diseased bees (Figure 4A). The expression trends of the 12 selected genes were consistent with the RNA-Seq results, which verified the reliability of the RNA-Seq data (Figure 4B).

## 4. Discussion

“Disease enters by the mouth”: most studies claim that pathogenic substances, such as bacterial, fungi, virus, and toxic chemicals, enter the honeybee via the digestive tract and first destroy intestinal morphology or microbial diversity, and then invade other tissues [13,14,15]. Therefore, the midgut of the honeybee is a vital tissue involved in the response to diseases [16]. In this study, we found that in honeybees with jujube flower disease, the midgut exhibited thinner cell layers and the peritrophic membrane was completely detached, suggesting that ingestion of jujube nectar (pollen) caused significant damage to the honeybee midgut. The peritrophic membrane is a natural barrier for midgut cells against pathogenic microorganisms and adverse chemical factors [17,18]. Insect chitinases are a group of important chitinolytic enzymes that hydrolyze the cuticle and peritrophic membranes of insects [19,20]. Chitinase activity in the midgut of DG honeybees was significantly higher than that of HG honeybees. We speculate that after the occurrence of jujube flower disease, chitinous substances in the periscopal membrane are degraded by chitinases, resulting in the destruction or even shedding of the periscopal membrane in the midgut. This eventually leads to digestion and absorption dysfunction in honeybees.

Among the theories on aging in social insects, particularly in *A. mellifera*, the most relevant to differential longevity in workers is the oxidative stress theory of aging, which posits that the irreversible accumulation of oxidative damage leads to senescence. Antioxidant enzymes are important defense enzymes in honeybees that can effectively scavenge superoxide radicals generated during metabolism and protect cells from oxidative damage [21]. SOD is a natural scavenger of oxygen-free radicals, and its level in vivo is an intuitive indicator of aging and death. CAT homogenously catalyzes the decomposition of H_2_O_2_ into H_2_O and O_2_, thereby protecting the body tissues from damage [22]. In this study, we found that the activities of SOD and CAT in the midgut of honeybees with jujube flower disease were significantly lower than those in the midgut of healthy honeybees, indicating that jujube flower disease can inhibit antioxidant capacity and disrupt the antioxidant system in the midgut, which could shorten honeybee lifespans.

The detoxification of exogenous substances in honeybees mainly depends on the levels of detoxification enzymes in vivo, among which the most important are CarE, GST, and CYP450 [23]. CarE mainly exists in the head and midgut of honeybees and catalyzes the hydrolysis of exogenous toxic substances into the body. CarE activity in the midgut of diseased honeybees was significantly higher than that in the midgut of healthy bees. It has been suggested that some toxic or harmful substances in the nectar or pollen of jujube flowers may induce an increase in CarE activity to protect the midgut from damage. However, both the CYP450 content and GST activity in the midgut of diseased honeybees were significantly decreased. In general, jujube flower disease has a significant negative impact on the detoxification ability of the midgut of bees, making it impossible to avoid damage caused by exogenous substances to the midgut. Constitutive quantitative changes in the expression of one or more P450 genes are among the most common mechanisms underlying insect resistance to xenobiotics. Our study found that ingestion of jujube nectar (pollen) upregulated CYP6a14/6a17 and downregulated CYP6a13/304a1 in the honeybee midgut, which is consistent with the above theory.

CYP450s perform various important physiological functions. In addition to detoxification metabolism, some CYP450 family genes in honeybees mediate the synthesis and metabolism of endogenous compounds, such as juvenile hormones, ecdysone, and maxillary gland secretions [24]. However, the most essential role of these genes in living organisms is in various biochemical processes as a key enzyme involved in oxidative reactions [25]. Unexpectedly, four CYP450 family DEGs were enriched in the GO term oxidoreductase activity, indicating that jujube flower disease greatly affects redox reactions in bees and causes metabolic disorders.

Finally, GO and KEGG enrichment analyses revealed the importance of two common DEGs, *AADC* and *AGXT2*. AADCs are homologous pyridoxal-5′-phosphate (PLP, active form of vitamin B6)-dependent enzymes that catalyze the conversion of aromatic L-amino acids into aromatic monoamines, mainly including the neurotransmitters dopamine, serotonin, and tyramine [26,27]. The accumulation of neurotransmitters causes overactivation of neurons, leaving the individual in a constant state of excitement, and even has a toxic effect on individual health. Our results showed that the expression of *AADC* was upregulated under the influence of jujube flower disease, which may explain why honeybees with jujube flower disease continued moving in circles around the beehive. AGXT2 is a class III pyridoxal-phosphate-dependent mitochondrial aminotransferase that affects lipid metabolism by elevating or reducing other substrates of this enzyme [28,29]. Moreover, the overexpression of AGXT2 protects against asymmetric dimethylarginine-induced endothelial dysfunction and aortic remodeling [30]. In this study, we found that *AGXT2* expression was significantly lower in honeybees with jujube flower disease, suggesting that jujube flower disease not only affects the digestive system but also has an impact on the circulatory system of honeybees. Thus, AGXT2 may be an attractive therapeutic target for the treatment of jujube flower disease.

## 5. Conclusions

Our study focused on the midgut, a vital component of the digestive system in honeybees, which serves as an interface between ingested food and the physiology of the insect and plays a pivotal role in nutrient absorption and immune defense mechanisms. We employed histopathological observations, enzyme activity assays, and transcriptomic methods to elucidate the effects of jujube flower disease on honeybee midguts, which revealed significant findings. Effects of jujube flower disease on the midgut included disruption of the peritrophic membrane, induction of chitin degradation, and interference with oxidative stress and immune defense. Transcriptomic analysis revealed that the genes differentially expressed between honeybees with jujube flower disease and healthy ones were related to oxidoreductase activity and vitamin binding. Moreover, two key DEGs, *AADC* and *AGXT2*, were closely associated with the occurrence of jujube flower disease, contributing to the underlying pathogenic mechanism of jujube flower disease in honeybees. We believe that these results will provide a reference for further research and for exploitation of the effective diagnosis and treatment of jujube flower disease.

## Figures and Tables

**Table 1:** Ten significantly enriched GO terms of the differentially expressed genes.

Category	ID	GO Description	*p*-Value	Upregulated Genes	Downregulated Genes
Molecular Function	GO:0005506	iron ion binding	0.001551	cytochrome P450 6a17 (LOC412209) cytochrome P450 6a14 (LOC112935903)	cytochrome P450 6a13 (LOC112939925) cytochrome P450 304a1 (LOC724175)
	GO:0016705	oxidoreductase activity, acting on paired donors, with incorporation or reduction of molecular oxygen	0.001905	cytochrome P450 6a17 (LOC412209) cytochrome P450 6a14 (LOC112935903)	cytochrome P450 6a13 (LOC112939925) cytochrome P450 304a1 (LOC724175)
	GO:0020037	heme binding	0.002311	cytochrome P450 6a17 (LOC412209) cytochrome P450 6a14 (LOC112935903)	cytochrome P450 6a13 (LOC112939925) cytochrome P450 304a1 (LOC724175)
	GO:0046906	tetrapyrrole binding	0.002459	cytochrome P450 6a17 (LOC412209) cytochrome P450 6a14 (LOC112935903)	cytochrome P450 6a13 (LOC112939925) cytochrome P450 304a1 (LOC724175)
	GO:0048037	cofactor binding	0.009924	cytochrome P450 6a17 (LOC412209) aromatic-L-amino-acid decarboxylase (LOC410638) cytochrome P450 6a14 (LOC112935903)	cytochrome P450 6a13 (LOC112939925) cytochrome P450 304a1 (LOC724175) Alanine-glyoxylate aminotransferase 2 (LOC408817)
	GO:0030170	pyridoxal phosphate binding	0.039579	aromatic-L-amino-acid decarboxylase (LOC410638)	Alanine-glyoxylate aminotransferase 2 (LOC408817)
	GO:0070279	vitamin B6 binding	0.039579	aromatic-L-amino-acid decarboxylase (LOC410638)	Alanine-glyoxylate aminotransferase 2 (LOC408817)
	GO:0019842	vitamin binding	0.046380	aromatic-L-amino-acid decarboxylase (LOC410638)	Alanine-glyoxylate aminotransferase 2 (LOC408817)
Biological Process	GO:0007155	cell adhesion	0.017147	Vinculin (LOC552082)	Nidogen-2 (LOC408797)
	GO:0022610	biological adhesion	0.017147	Vinculin (LOC552082)	Nidogen-2 (LOC408797)

**Table 2:** Metabolism pathway enrichment analysis of the differentially expressed genes.

KEGG ID	Pathway Name	*p*-Value	Upregulated Genes	Downregulated Genes
ame00130	Ubiquinone and other terpenoid-quinone biosynthesis	0.099986	—	4-coumarate--CoA ligase 1 (LOC726625)
ame00350	Tyrosine metabolism	0.107270	Aromatic-L-amino-acid decarboxylase (LOC410638)	—
ame00760	Nicotinate and nicotinamide metabolism	0.128784	—	Purine nucleoside phosphorylase (LOC408299)
ame00380	Tryptophan metabolism	0.163549	Aromatic-L-amino-acid decarboxylase (LOC410638)	—
ame00240	Pyrimidine metabolism	0.210006	—	Purine nucleoside phosphorylase (LOC408299)
ame00270	Cysteine and methionine metabolism	0.283985	—	Methylthioribose-1-phosphate isomerase (LOC409023)
ame00010	Glycolysis/Gluconeogenesis	0.318452	Multiple inositol polyphosphate phosphatase 1 (LOC409751)	—
ame00564	Glycerophospholipid metabolism	0.392835	—	Alanine--glyoxylate aminotransferase 2 (LOC408817)
ame00562	Inositol phosphate metabolism	0.318452	Multiple inositol polyphosphate phosphatase 1 (LOC409751)	—
ame00230	Purine metabolism	0.110121	—	Purine nucleoside phosphorylase (LOC408299) Bifunctional 3′-phosphoadenosine 5′-phosphosulfate synthase 2 (LOC408299)

## Data Availability

Data are contained within the article and Supplementary Materials.

## Supplementary Materials

The following supporting information can be downloaded at: https://www.mdpi.com/article/10.3390/genes15050533/s1, Table S1: The information of primers in qRT-PCR; Table S2: Statistics for filtering and mapping reads; Table S3: Differentially expressed genes between HG and DG; Table S4: GO enrichment analysis of the differentially expression genes between HG and DG; Table S5: KEGG enrichment analysis of the differentially expression genes between HG and DG.
